# Metabolic remodeling and calcium handling abnormality in induced pluripotent stem cell-derived cardiomyocytes in dilated phase of hypertrophic cardiomyopathy with MYBPC3 frameshift mutation

**DOI:** 10.1038/s41598-024-62530-0

**Published:** 2024-07-04

**Authors:** Haruka Mori, Dongzhu Xu, Yuzuno Shimoda, Zixun Yuan, Yoshiko Murakata, Binyang Xi, Kimi Sato, Masayoshi Yamamoto, Kazuko Tajiri, Tomoko Ishizu, Masaki Ieda, Nobuyuki Murakoshi

**Affiliations:** 1 Department of Cardiology, Faculty of Medicine, University of Tsukuba, 1-1-1 Tennoudai, Tsukuba, Ibaraki 305-8575 Japan; 2 Master’s Program in Medical Sciences, University of Tsukuba, Tsukuba, Japan; 3 Department of Cardiology, National Cancer Center Hospital East, Kashiwa, Japan; 4 Department of Cardiology, Keio University School of Medicine, Tokyo, Japan

**Keywords:** Dilated phase of hypertrophic cardiomyopathy, Induced pluripotent stem cells, Myosin binding protein C, Cardiomyocytes, Energy metabolism, Cardiomyopathies, Experimental models of disease, Induced pluripotent stem cells

## Abstract

Hypertrophic cardiomyopathy (HCM) is an inherited disorder characterized by left ventricular hypertrophy and diastolic dysfunction, and increases the risk of arrhythmias and heart failure. Some patients with HCM develop a dilated phase of hypertrophic cardiomyopathy (D-HCM) and have poor prognosis; however, its pathogenesis is unclear and few pathological models exist. This study established disease-specific human induced pluripotent stem cells (iPSCs) from a patient with D-HCM harboring a mutation in *MYBPC3* (c.1377delC), a common causative gene of HCM, and investigated the associated pathophysiological mechanisms using disease-specific iPSC-derived cardiomyocytes (iPSC-CMs). We confirmed the expression of pluripotent markers and the ability to differentiate into three germ layers in D-HCM patient-derived iPSCs (D-HCM iPSCs). D-HCM iPSC-CMs exhibited disrupted myocardial sarcomere structures and an increased number of damaged mitochondria. Ca^2+^ imaging showed increased abnormal Ca^2+^ signaling and prolonged decay time in D-HCM iPSC-CMs. Cell metabolic analysis revealed increased basal respiration, maximal respiration, and spare-respiratory capacity in D-HCM iPSC-CMs. RNA sequencing also showed an increased expression of mitochondrial electron transport system-related genes. D-HCM iPSC-CMs showed abnormal Ca^2+^ handling and hypermetabolic state, similar to that previously reported for HCM patient-derived iPSC-CMs. Although further studies are required, this is expected to be a useful pathological model for D-HCM.

## Introduction

Hypertrophic cardiomyopathy (HCM) is an inherited cardiac disorder characterized by ventricular wall hypertrophy and diastolic dysfunction. It is diagnosed by excluding secondary cardiomyopathies that result from various systemic diseases. In about 40–60% of cases, pathogenic mutations are found in genes encoding myocardial component proteins, primarily sarcomeres^1,2^. *MYBPC3*, which encodes cardiac myosin-binding protein C (cMYBP-C), is the most frequent causative gene for HCM along with *MYH7,* a β-myosin heavy chain-coding gene^2^. More than 350 *MYBPC3* mutations that can cause HCM have been identified, and various *MYBPC3* mutations have been identified in all regions^3,4^. The penetrance of HCM caused by *MYBPC3* mutations increases with age, and the average penetrance in all generations is approximately 60%^5^. In HCM caused by *MYH7* mutation, increased Ca^2+^ sensitivity, concomitant increased muscle contractility, and decreased muscle relaxation are present. Mutant sarcomere proteins often exhibit impaired Ca^2+^-dependent actomyosin cross-bridge cycling, altered Ca^2+^ sensitivity, and impaired force generation^6^. A similar mechanism has been estimated for HCM caused by *MYBPC3* mutations, and haploinsufficiency has also been estimated as a mechanism of HCM pathogenesis because *MYBPC3* mutations are mostly truncated^7^. However, the involvement of *MYBPC3* mutations in the pathogenesis of HCM is not fully understood^7–9^.

Most patients with HCM have a relatively gradual disease course or may be asymptomatic; however, some progress to the dilated phase of hypertrophic cardiomyopathy (D-HCM), in which the left ventricular wall becomes thin and the ventricular chamber becomes large, resulting in systolic dysfunction^10,11^. D-HCM can cause heart failure, arrhythmias, and sudden death. Its symptoms, clinical course, and treatment are similar to that of dilated cardiomyopathy (DCM). However, D-HCM has a poorer prognosis than DCM and there is no fundamental treatment other than heart transplantation^12,13^. Moreover, there are few reports on the causes and pathological mechanisms of D-HCM in cellular and animal models.

In this study, we generated disease-specific human induced pluripotent stem cells (iPSCs) from a patient with *MYBPC3* truncating mutation and analyzed the molecular cellular characteristics of disease-specific iPSC-derived cardiomyocytes (iPSC-CMs). This study aimed to explore the molecular mechanisms underlying the pathogenesis of D-HCM.

## Results

### Patient characteristics

**Figure 1:**
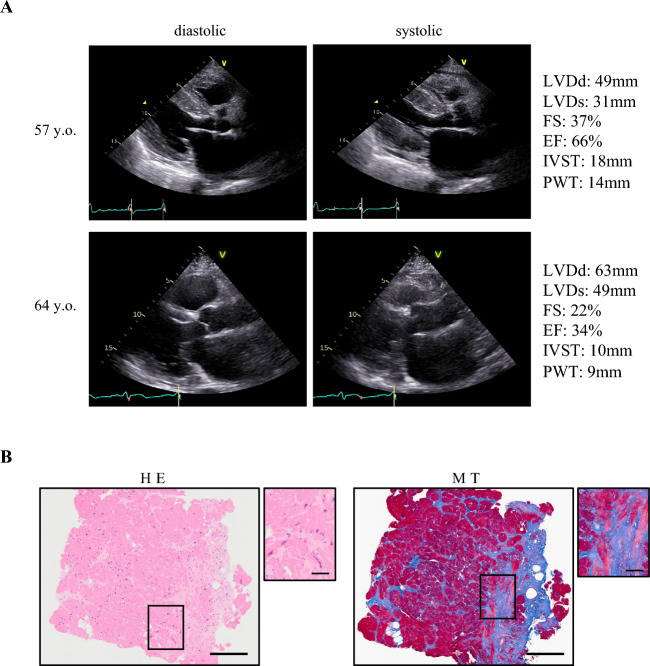
Echocardiograms and pathological images in a patient with D-HCM. (**A**). Diastolic (left) and systolic (right) echocardiograms at 57 (above) and 64 years old (below), respectively. LVDd: left ventricular end-diastolic diameter, LVDs: left ventricular end-systolic diameter, EF: ejection fraction, IVST: interventricular septal thickness, PWT: posterior LV wall thickness. (**B**). Histological staining of endomyocardial biopsy sample: Hematoxylin–eosin stain (HE: left) and Masson trichrome stain (MT: right) (Scale: 200 µm (Full), 50 µm (Enlarged)).

The patient was diagnosed with impaired glucose tolerance approximately at age 50 years and was diagnosed with atrial fibrillation at 53 years. The patient’s father was diagnosed with and treated for heart failure and atrial fibrillation; however, other detailed information was unknown. At 57 years, edema and shortness of breath appeared when using the stairs. Echocardiography showed asymmetric interventricular septal hypertrophy with normal ventricular dimensions and an ejection fraction (EF) (EF 66%) (Fig. 1A). Based on the results of the cardiac catheterization, the patient was diagnosed with HCM. At the age of 60 years, he developed ventricular fibrillation and underwent cardioverter-defibrillator implantation. Subsequently, a decrease in left ventricular systolic function and ventricular dilatation were observed (EF 34%) (Fig. 1A). Genetic analysis using panel sequencing revealed a frameshift mutation (c.1377delC, p.L460Wfs*5) in *MYBPC3*, but no other pathogenic variants. Histological staining of the endomyocardial biopsy samples revealed myocardial degeneration and severe fibrosis (Fig. 1B). Based on clinical findings and genetic analysis, the patient was diagnosed with D-HCM. The patient underwent standard medical therapy for heart failure, and multiple catheter ablation procedures for atrial fibrillation and ventricular tachycardia. However, the patient’s health condition progressively worsened and he died of heart failure at the age of 65 years.

### Generation of D-HCM iPSCs

**Figure 2:**
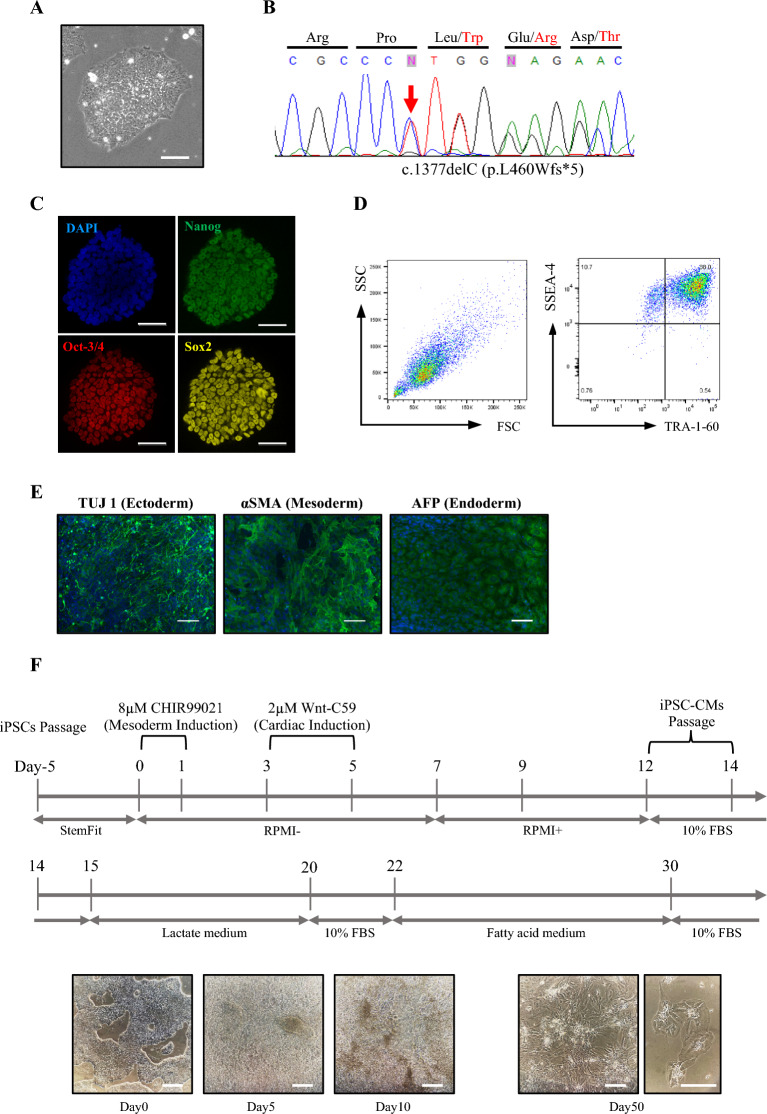
Pluripotency of the D-HCM iPSCs and cardiac differentiation of iPSCs into iPSC-CMs. (**A**). D-HCM iPSCs form several colonies (scale,100 µm). (**B**). DNA sequencing analysis of D-HCM iPSCs reveals *MYBPC3* mutation c.1377delC (red arrow). (**C**). Immunofluorescence with three pluripotency markers (Nanog, Oct3/4, and Sox2) (scale:50 µm). (**D**). Flow cytometry of two pluripotency markers (SSEA-4 and TRA-1–60). (**E**). Immunofluorescence with three germ layer markers: ectoderm (TUJ1), mesoderm (αSMA), endoderm (AFP) (Scale:100 µm). (**F**). Cardiac induction was performed using the GiWi protocol (Day0-14), purification with a lactate medium (Day15-20), and maturation with a fatty acid medium (Day22-30). In the lower part, photographs show each stage of cardiac differentiation on Day 0, Day 5, Day 10, and Day 50 (Scale:200 µm).

The established D-HCM iPSCs formed colonies and proliferated as shown in Fig. 2A. Direct DNA sequencing of D-HCM iPSCs revealed the same mutation (c.1377delC) in *MYBPC3* as that in the donor blood sample (Fig. 2B). iPSCs were highly stained for the pluripotency markers Nanog, Oct3/4, and Sox2 (Fig. 2C). Flow cytometry using the stem cell markers SSEA-4 and TRA-1-60 showed that 88.0% of D-HCM iPSCs were positive for both markers (Fig. 2D). To confirm whether D-HCM iPSCs had differentiation potential in all three germinal directions, we performed immunofluorescence using the germ markers TUJ1 (ectoderm), SMA (mesoderm), and AFP (endoderm). The D-HCM iPSCs stained positive for all three germ markers. (Fig. 2E). From these results, we confirmed the generation of disease-specific iPSCs from the PBMCs of a D-HCM patient with the *MYBPC3* mutation (c.1377delC).

### Cardiac characteristics in iPSC-CMs

**Figure 3:**
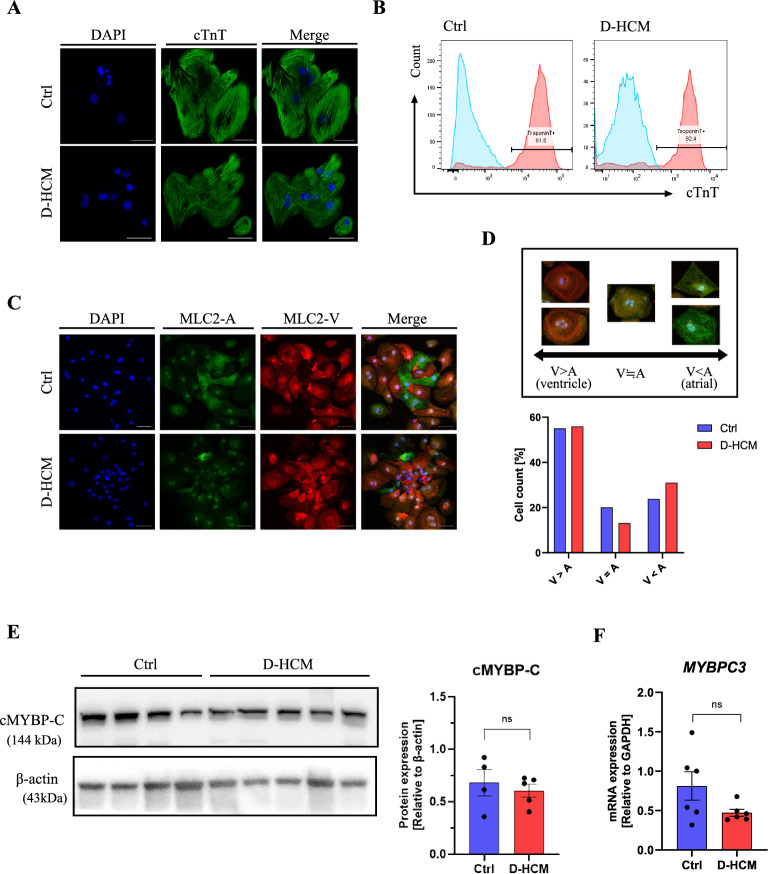
Cardiac characteristics of iPSC-CMs. (**A**). Immunofluorescence for Cardiac Troponin T (cTnT) (Scale: 50 µm). (**B**). Flow cytometry with cTnT (blue wave: negative control, red wave: cTnT positive). (**C**). Immunofluorescence for MLC2-A and MLC2-V (Scale: 50 µm). blue: DAPI, green: MLC2-A, red: MLC2-V. (**D**). Fluorescence-positive cell count (%) for MLC2-A and MLC2-V in Fig. 2C. iPSC-CMs were classified into three groups according to the expression levels, V > A, V≒A, V < A (V: MLC2-V, A: MLC2-A). (**E**). Protein expression level of cMYBP-C (Ctrl: N = 4 from 2 cell lines, D-HCM: N = 5 from 1 cell line). (**F**). mRNA expression level of *MYBPC3* (Ctrl: N = 6 from 2 cell lines, D-HCM: N = 6 from 1 cell line).

Autonomous beating was observed approximately one week after cardiac induction (Fig. 2F). Cardiac sarcomere structures of iPSC-CMs were observed using fluorescent immunostaining for cardiac troponin T (cTnT) (Fig. 3A). Flow cytometry revealed that more than 80% of the control- (Ctrl) and D-HCM iPSC-CMs were positive for cTnT (Fig. 3B). The maturity of iPSC-CMs was assessed by fluorescent immunostaining for MLC2-A (immature and atrial muscle markers) and MLC2-V (ventricular muscle marker). Generally, MLC2-V is selectively expressed in ventricular CMs, whereas MLC2-A is expressed in both atrial and ventricular CMs. Moreover, the expression of MLC2-A in immature ventricular CMs gradually decreased with CMs maturation^14^. Although some iPSC-CMs co-expressed MLC2-A and MLC2-V, more than 50% of iPSC-CMs were MLC2-V dominant (Ctrl:55.0%; D-HCM:55.9%) (Fig. 3C,D). c-MYBP-C protein and *MYBPC3* mRNA expression levels in iPSC-CMs were not significantly different between the control and D-HCM groups (Fig. 3E,F). Moreover, troponin I (TnI) protein expression was also not significantly different between the control and D-HCM groups (Supplementary Figure S1).

### Increased myocardial sarcomere disarray and mitochondrial damage in D-HCM iPSC-CMs

**Figure 4:**
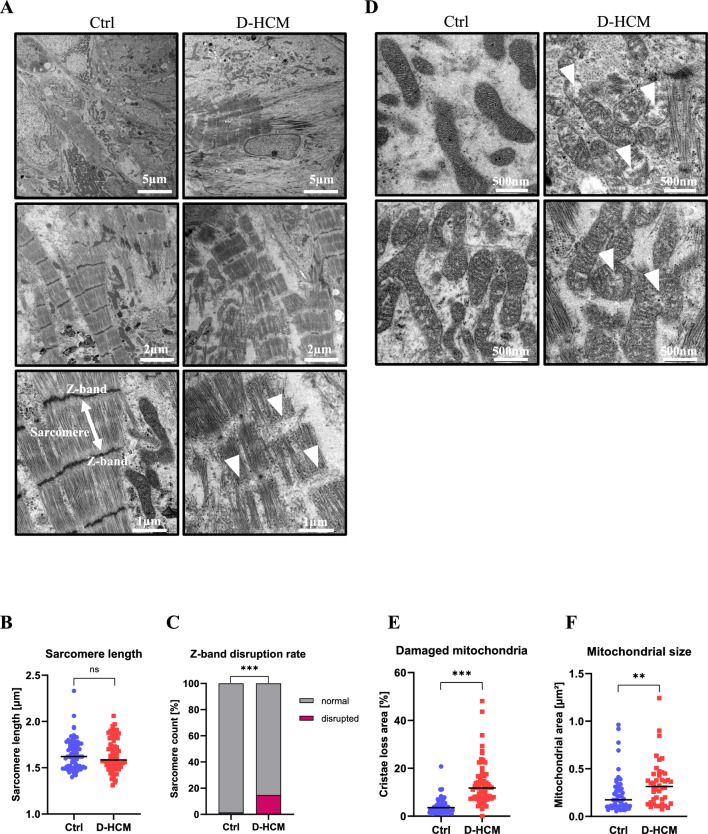
Electron microscopy images of cardiac sarcomeres and mitochondria in iPSC-CMs. (**A**). Cardiac sarcomere structures. Z-band disruption and sarcomere disarray were observed in D-HCM iPSC-CMs (white arrow) (Scale: 5 µm (upper), 2 µm (middle), 1 µm (lower)). (**B**). Sarcomere lengths (µm) (Ctrl: N = 71, D-HCM: N = 72). (**C**). Z-band disruption rate (%) measured by sarcomere units (Ctrl: N = 140, D-HCM: N = 220). “N” represents the sarcomere units in A, B, and C. (**D**). Mitochondrial structures. More damaged mitochondria were observed in D-HCM iPSC-CMs (white arrow) (Scale: 500 nm). (**E**). The cristae loss area (%) indicates damaged mitochondria (Ctrl: N = 72, D-HCM: N = 68). (**F**). Mitochondrial size (um^2^) (Ctrl: N = 53, D-HCM: N = 42). “N” represents the numbers of mitochondria in D, E, and F. All results of TEM were obtained from Ctrl 1 cell line and D-HCM 1 cell line. n.s. indicates not significant vs. Ctrl; **p < 0.01 vs. Ctrl; ***p < 0.001 vs. Ctrl.

The intracellular myocardial structures of iPSC-CMs were evaluated using TEM, and cardiac sarcomere structures were clearly observed in both the Ctrl- and D-HCM groups (Fig. 4A). Most sarcomeres were regularly arranged in the Ctrl group, whereas some sarcomeres in the D-HCM group showed Z-band disruption and sarcomere disarray (Fig. 4A, white arrows). Sarcomere length did not differ between the Ctrl group (1.65 ± 0.02 μm) and the D-HCM group (1.64 ± 0.02 μm) (Fig. 4B). The Z-band disruption rate, measured in sarcomere units, was 1.4% in the Ctrl group and 14.7% in the D-HCM group (Fig. 4C), and this difference was statistically significant. Next, we evaluated the mitochondrial structure in iPSC-CMs. Cristae are internal mitochondrial structures and its loss indicates mitochondrial damage^15^. As shown in Fig. 4D, cristae loss area per mitochondrion was significantly higher in D-HCM (13.6 ± 1.1%) than in the Ctrl group (4.2 ± 0.37%) (Fig. 4E). Additionally, the mitochondrial area was larger in the D-HCM (0.35 ± 0.04 μm^2^) than in the Ctrl group (0.24 ± 0.03 μm^2^) (Fig. 4F).

### Increased abnormal Ca^*2*+^ transients in D-HCM iPSC-CMs

**Figure 5:**
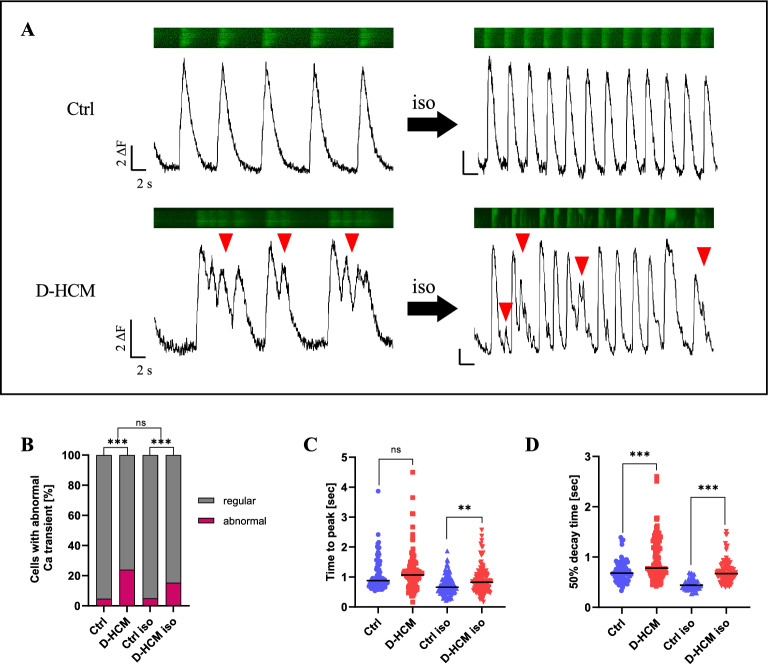
Intracellular Ca^2+^imaging in iPSC-CMs. (**A**). Ca^2+^ transients before and after isoproterenol (iso) treatment. Abnormal Ca^2+^ transients were increased in D-HCM iPSC-CMs (red arrow). (**B**). Number of cells with abnormal Ca^2+^ transients (%). N = 21 (Ctrl), N = 25 (D-HCM), N = 20 (Ctrl iso), N = 26 (D-HCM iso).　“N” represents the number of cells. (**C**). Time to peak (second: sec) (**D**). 50% decay time (sec). n.s. indicates not significant vs. Ctrl; **p < 0.01 vs. Ctrl; ***p < 0.001 vs. Ctrl.

The representative Ca^2+^ signals and transients are shown in Fig. 5A. Compared to Ctrl iPSC-CMs, abnormal Ca^2+^ signals (early after depolarization (EAD) and delayed after depolarization (DAD)) increased in the D-HCM iPSC-CMs both before and after 1 µM isoproterenol (iso) treatment. The percentages of cells with abnormal Ca^2+^ transients were 4.8% in the Ctrl, 24.0% in the D-HCM, 5.0% in the Ctrl treated with iso, and 15.4% in the D-HCM treated with iso (Fig. 5B). The peak time was prolonged in D-HCM iPSC-CMs after iso treatment (Fig. 5C), and the decay time was significantly prolonged in D-HCM iPSC-CMs with or without iso treatment (Fig. 5D).

### Increased mitochondrial energy production and metabolic shift beginning in D-HCM iPSC-CMs

**Figure 6:**
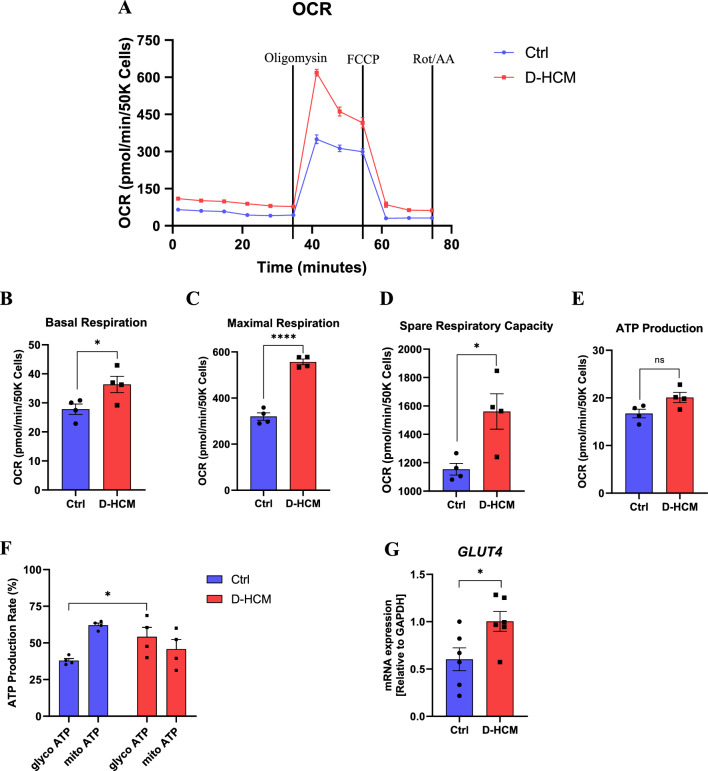
Mitochondrial and glycolytic energy production in iPSC-CMs. (**A**). Cell Mito Stress Test (blue: Ctrl, red: D-HCM). Black lines indicate the addition of mitochondrial functional modulators (Oligomycin, FCCP, Rotenone/Antimycin A). Vertical axis: oxygen consumption rate (OCR), horizontal axis: time. (**B–E**). Energy production indicators in mitochondrial respiration calculated from Fig. 5A. (**B**). Basal respiration. (**C**). Maximal respiration. (**D**). Spare respiratory capacity. (**F**). Mitochondrial and glycolytic ATP production rate measured by Real-Time ATP Rate Assay. The left graph shows the ATP production rate (pmol/min/50 K Cells). The right graph shows the ATP production rate (%). glyco ATP: glycolytic ATP production (anaerobic respiration), mito ATP: mitochondrial ATP production (aerobic respiration). (**G**). mRNA expression levels of *GLUT4* by RT-qPCR. n.s. indicates not significant vs. Ctrl; *p < 0.05 vs. Ctrl; ***p < 0.001 vs. Ctrl.

The mitochondrial stress test was used to assess mitochondrial aerobic respiration (Fig. 6A), and a Real-Time ATP Rate Assay was used to assess the ratio of aerobic to anaerobic respiration in ATP production. Basal respiration, maximal respiration, and spare respiratory capacity significantly increased in D-HCM iPSC-CMs, suggesting accelerated mitochondrial energy production (Fig. 6B,C,D). ATP production tended to be higher in D-HCM iPSC-CMs; however, this difference was not statistically significant (Fig. 6E). Real-time ATP rate assays showed that mitochondrial ATP production was predominant in Ctrl iPSC-CMs, whereas glycolytic ATP production was predominant in D-HCM iPSC-CMs and was significantly higher than that in the Ctrl group (Fig. 6F). RT-qPCR showed that the expression of the glucose transporter *GLUT4* was upregulated in D-HCM iPSC-CMs (Fig. 6G). These results suggested increased mitochondrial energy production and the beginning of the metabolic shift to glycolysis in D-HCM iPSC-CMs.

### Upregulated mitochondria-related gene expressions in D-HCM iPSC-CMs

**Figure 7:**
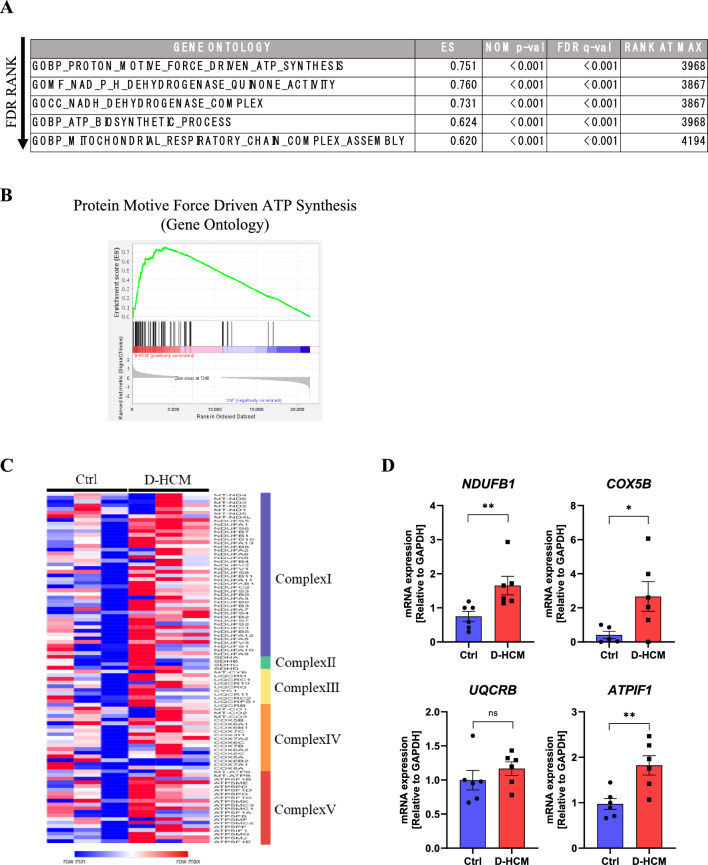
Gene expression pattern in D-HCM iPSC-CMs. (**A**). GSEA analysis using Gene Ontology (GO). Top-ranked gene set lists were arranged in each table (*p* < 0.05 and low FDR rank). Ctrl: N = 4 from 2 cell lines, D-HCM: N = 5 from 1 cell line. (**B**). Graphs of the top two gene sets in GO. (**C**). Heatmap of mitochondrial electron transport-related genes. (**D**). mRNA expression levels of mitochondrial electron transport-related genes: *NDUFB1, COX5B, UQCRB* and *ATPIF1* by RT-qPCR. n.s. indicates not significant vs. Ctrl; *p < 0.05 vs. Ctrl; **p < 0.01 vs. Ctrl.

RNA sequencing was performed to examine the differences in gene expression patterns between Ctrl and D-HCM iPSC-CMs. According to enrichment analysis using the GSEA software^16^, mitochondria-related gene expression was enriched in D-HCM iPSC-CMs. In GO analysis, the top-ranked gene sets in D-HCM iPSC-CMs were closely related to ATP production and mitochondrial metabolism (Fig. 7A,B). The heatmap in Fig. 7C shows that gene expression of the electron transport system Complex I-V in the mitochondria was elevated in D-HCM iPSC-CMs. Among the mitochondrial electron transport system-related genes, the expression levels of *NDUFB1*, *COX5B* and *ATPIF1* were significantly increased in D-HCM iPSC-CMs, as confirmed by RT-qPCR (Fig. 7D).

## Discussion

In this study, we established iPSCs from PBMCs of a D-HCM patient carrying *MYBPC3* truncating mutation (c.1377delC), compared the phenotypes of Ctrl iPSC-CMs and D-HCM iPSC-CMs generated by cardiac differentiation and obtained the following findings: (1) TEM showed that D-HCM iPSC-CMs had a more disorganized sarcomere structure and intramitochondrial cristae defects. (2) Ca^2+^ imaging showed that D-HCM iPSC-CMs exhibited more abnormal Ca^2+^ signals and longer decay times. (3) Cell metabolic analysis showed that mitochondrial energy production increased, and energy metabolism began to switch to glycolytic dominance in D-HCM iPSC-CMs. Furthermore, (4) RNA-seq and RT-qPCR showed that the expression levels of oxidative phosphorylation- and ATP production-related genes were upregulated in D-HCM iPSC-CMs. This is the first study to show the pathophysiological phenotypes of disease-specific iPSC-CMs established from a patient with D-HCM and may be a useful tool for elucidating the pathogenesis of D-HCM and developing new therapeutic strategies.

c-MYBP-C binds to myosin and actin, regulates their positions, and plays a role in maintaining normal cardiac sarcomere structure and regulating normal contractions^3,8,17^. All C0-10 domains of cMYBP-C are required for normal cardiac function, and various pathogenic mutations in each domain have been reported as the causative genes of HCM. The *MYBPC3* c.1377delC mutation identified in this study resulted in a frameshift and truncated protein, p.L460Wfs*5 (located in the C3 domain^17^), which has previously been reported as a causative gene mutation in HCM. This variant has not been identified in the general population and is presumed to result in an absent or nonfunctional protein product, although functional assays have not been conducted. Although D-HCM-specific pathogenic variants are unknown, previous studies have reported that some *MYBPC3* pathogenic variants, such as p.R945fs and p.R820Q, are detected in patients with D-HCM^18,19^. Pathogenic mutations in *MYBPC3* are presumably responsible for impaired myosin-actin binding and abnormal cardiac sarcomere structures resulting in excessive contraction^20–22^. Disarrangement of the sarcomere structure has also been observed in patients with HCM carrying mutations in other sarcomere genes such as *MYH7*, which encodes the cardiac myosin heavy chain, and may be a common finding in HCM with sarcomere-related gene mutations^23–25^. Most *MYBPC3* mutations that cause HCM are of the short form, and haploinsufficiency has been suggested as a mechanism of pathogenesis^7^. In this study, *MYBPC3* mRNA and protein levels were not significantly different between Ctrl iPSC-CMs and D-HCM iPSC-CMs. Previous studies have reported that *MYBPC3* mRNA and protein expression is decreased or unchanged in iPSC-CMs with *MYBPC3* mutations, depending on the type and site of *MYBPC3* mutations^7,26,27^.

In this study, the size of D-HCM iPSC-CMs was not larger than that of Ctrl iPSC-CMs. A previous study reported that the cell size was indeed larger in HCM iPSC-CMs with *MYBPC3* mutations than in Ctrl iPSC-CMs; however, in the presence of serum, cardiomyocyte hypertrophy was masked and rather smaller in HCM iPSC-CMs with *MYBPC3* mutation^28^. Another study also reported that HCM iPSC-CMs with *MYBPC3* mutations showed no significant difference in cell size compared to Ctrl iPSC-CMs^27^. This may be because iPSC-CMs mimic cardiomyocytes at the developmental stage and thus reflect the early prehypertrophic stage of HCM^29^.

In this study, Ca^2+^ imaging showed abnormal Ca^2+^ handling in D-HCM iPSC-CMs. The prolonged decay time of D-HCM iPSC-CMs may reflect diastolic dysfunction^24,25,30^, which is a characteristic clinical finding in HCM. In addition, the increased abnormal Ca^2+^ transients in D-HCM iPSC-CMs suggested triggered activity, including EAD and/or DAD, which are important mechanisms in the pathogenesis of arrhythmias. Intracellular Ca^2+^-handling abnormalities are common pathophysiological features of HCM observed in disease-specific iPSC-CMs, animal models, and human tissue samples, including markedly increased intracellular Ca^2+^ levels, prolonged Ca^2+^ transient, and triggered activity^31^. HCM-causing sarcomeric mutations are thought to increase myofilament Ca^2+^ sensitivity, myocardial contractility, and impair myocardial energetics, resulting in a hypermetabolic mitochondrial state and abnormal Ca^2+^ handling^32^.

In a previous study, increased basal respiration and ATP production were observed in genome-edited *MYH7* mutant HCM iPSC-CMs, which is consistent with our results^33^. In contrast, another report showed that ATP production decreased in myocardial samples from patients with HCM who underwent septal myectomy^15^. Although this discrepancy may be attributed to differences in the disease stage or the analyzed samples, the relative increase in energy demand seems to be a common feature of HCM^34^. The increased mitochondrial respiratory capacity of D-HCM iPSC-CMs observed in our cellular energy metabolism analysis was presumably due to a compensatory mechanism for abnormal sarcomeric functional changes and excess energy demand, which was predicted to increase oxidative stress and cause mitochondrial damage. This suggests that D-HCM iPSC-CMs exhibit a phenotype similar to HCM in terms of cellular energy metabolism^22^. In this study, cristae loss was frequently observed in D-HCM iPSC-CMs by TEM. Excessive mitochondrial energy production is predicted to increase oxidative stress and cause mitochondrial damage^15^. In addition, since most mitochondria in cardiomyocytes are arranged near sarcomeres and provide energy for myocardial contraction, it is possible that the alteration in sarcomere structures directly affects mitochondrial function via the mitochondrial network^35^. Furthermore, some reports have shown that the failure to upregulate mitophagy is partly responsible for the accumulation of damaged mitochondria in HCM^36,37^.

In this study, D-HCM iPSC-CMs showed elevated expression of glycolysis-related genes *GLUT4* and *ATPIF1* and glycolysis-dominant energy metabolism, suggesting metabolic remodeling of the D-HCM state. A recent study demonstrated that *ATPIF1* upregulation contributes to the switch from oxidative metabolism to glycolysis in the heart during the development of pathological hypertrophy, and that deletion of *ATPIF1* in cardiomyocytes or scavenging of mitochondrial ROS prevents the metabolic switch and protects against pathological remodeling^38^. Since the transition from oxidative fatty acid metabolism to glucose metabolism is thought to be associated with pathological remodeling of the heart^30^, the metabolic switch and accumulation of mitochondrial damage may contribute to the transition to the dilated phase in patients with HCM.

This study had some limitations. We used only one patient-derived iPSC-CM and compared it with iPSC-CMs from two unrelated healthy subjects as controls. In the future, it would be desirable to establish more iPSCs from patients with D-HCM and create isogenic controls for comparison. Although iPSC-CMs are useful and non-invasive tools for exploring pathological mechanisms and discovering novel therapeutic targets for heart diseases, cardiomyocyte immaturity is a limitation in the application of iPSC-CMs for research purposes^39,40^. In this study, we used the fatty acid medium method to accelerate iPSC-CM maturation^41^. The phenotype of D-HCM iPSC-CMs was similar to that of the pathological models of HCM reported in previous studies^22,23,25,26,33^. Therefore, we were unable to completely elucidate the precise molecular mechanisms of transition to D-HCM. Further studies are needed to elucidate the detailed pathophysiological mechanisms underlying D-HCM.

In conclusion, disease-specific iPSCs (D-HCM iPSCs) were established from a patient with D-HCM and a shortened *MYBPC3* mutation (c.1377delC). D-HCM iPSC-CMs showed an abnormal cardiac sarcomere structure, increased damaged mitochondria, abnormal Ca^2+^ transients, increased mitochondrial energy production, and upregulated glycolysis-related gene expression. D-HCM iPSC-CMs showed a phenotype similar to that of a previously reported pathological model of HCM; however, further studies are needed to elucidate the pathological mechanism of D-HCM.

## Methods

All experiments were performed in accordance with the latest version of the Declaration of Helsinki and the relevant guidelines and regulations including the Ethical Guidelines for Medical and Health Research Involving Human Subjects. The generation and experiments of iPSC were reviewed and approved by the University of Tsukuba Clinical Research Ethics Review Committee (approval number: R02-078). The study on the genetic analysis was also reviewed and approved by the University of Tsukuba Clinical Research Ethics Review Committee (approval number: R02-300).

### Generation of iPSC from a patient with D-HCM

iPSCs were generated as described in our previous paper^42^. Briefly, after obtaining written informed consent from the patient, the peripheral blood mononuclear cells (PBMCs) were collected from the patient’s peripheral blood through density gradient centrifugation using Ficoll-Plaque and were cultured with PBMC medium (Supplemental Table). After one week, Human iPS Cell Generation Episomal Vector Mix (TaKaRa) was transfected into the PBMCs by electroporation using the Neon Transfection System (Thermo Fisher Scientific), and cells were quickly spread onto the iMatrix-coated plates with StemFit medium (Supplemental Table) supplemented 10 µM Y-27632 (FUJIFILM Wako). About two weeks later, iPSC colonies were picked under a microscope, and D-HCM patient-derived iPSCs (D-HCM iPSCs) were established. Two cell lines, HPS3354 and HPS3386, were purchased from the RIKEN BioResource Center and used as healthy control cells (Ctrl iPSCs).

### Genetic analysis

After obtaining written informed consent from the patient, next-generation sequencing was performed with the Ion Proton System (Thermo Scientific, Waltham, Massachusetts, USA) using the Ion AmpliSeq™ Cardiovascular Research Panel and the Ion AmpliSeq™ Library Kit 2.0. Primary processing of reads was performed using the Ion Proton Software (Thermo Fisher Scientific). Alignment with the reference genome (GRCh38–hg19), coverage analysis, and variant calling were performed using standard parameters in the Ion Torrent Software Suite (ISS) version 5.4.0. The VCF file was uploaded and annotated using wANNOVAR software. Variants classified as pathogenic or likely pathogenic for DCM, HCM, and familial atrial fibrillation according to the ClinVar were considered as causative genes, which were confirmed by direct sequencing.

### Cardiac differentiation

Cardiac differentiation was performed according to the GiWi (GSK-3β (glycogen synthase kinase-3β) inhibitor and Wnt inhibitor) protocol^41,43^, and mediums and reagents are listed in the Supplemental Table. iPSCs were cultured with StemFit in a 5% CO_2_ incubator at 37℃. When the cells reached 80–90% confluence, the medium was replaced with RPMI (Supplemental Table) containing 8 µM CHIR99021 (a GSK3β inhibitor, Sigma-Aldrich). Twenty-four hours later (Day 1), the medium was replaced with RPMI, and the cells were cultured. Forty-eight hours later (Day 3), the medium was changed with RPMI- medium + 2 µM Wnt-C59 (an Wnt inhibitor, Cayman Chemical Company). On Day 5, the medium was replaced with RPMI- medium. On day 7, the medium was replaced with the RPMI + medium (Supplemental Table). After these processes, the medium was replaced with RPMI + every two days. During days 12–14, the cells were passaged on Matrigel-coated 6-well plates with 10% FBS medium (Supplemental Table) supplemented with 10 µM Y-27362. Two days later, the medium was replaced with lactate (Supplemental Table) to remove non-iPSC-CMs. Five days later, the medium was replaced with 10% FBS medium. Two or three days later, the medium was replaced with fatty acid medium (Supplemental Table) for cell maturation. Approximately one week later, the medium was replaced with 10% FBS medium, and the medium was replaced every three days. iPSC-CMs were used in our experiments approximately 50 days after cardiac differentiation (Days 45–55).

### Immunofluorescence

Immunofluorescence analysis was performed as previously described^42^. Briefly, cells on a Matrigel-coated cover glass were fixed with 4% paraformaldehyde and permeabilized with 0.1% Triton X-100 (Sigma-Aldrich). After washing, cells were blocked with 1% BSA. The primary antibody was reacted overnight at 4 °C. The next day, the cells on the cover glass were incubated with a secondary antibody for one hour at room temperature. After washing, nuclear staining was performed, and the samples were sealed. The images were acquired using a confocal laser microscope TCS SP8 (Leica Microsystems). The primary antibodies used were a Human Pluripotent Stem Cell 3-Color Immunocytochemistry Kit (R&D Systems), Anti-Cardiac Troponin T antibody (1:200, Richard-Allan Scientific), Anti-MLC-2 V antibody (1:200, Proteintech), and anti-MLC-A antibody (1:200, Synaptic Systems). Secondary antibodies used were Alexa Fluor 488 goat anti-mouse IgG (1:2000) and Alexa Fluor 555 goat anti-rabbit IgG (1:2000; Thermo Fisher Scientific). DAPI (300 nM) and FluoroPure (Thermo Fisher Scientific) were used for nuclear staining.

### Flow cytometry

Flow cytometry was performed as described previously^42^. Briefly, the collected cells were stained using the Live/Dead Fixable Dead Cell Stain Kit (1:1000; Thermo Fisher Scientific). The samples were fixed and permeabilized using Foxp3/Transcription Factor Fixation/Permeabilization Concentrate and Diluent (Thermo Fisher Scientific), respectively. The primary antibody was diluted in permeabilization buffer and incubated for 30 min at room temperature. After washing, the cells were incubated with secondary antibody for 20 min at room temperature. The samples were suspended in an auto-MACS buffer and passed through a filter. The samples were analyzed using the BD FACSVerse System (BD Biosciences) and FlowJo (BD Biosciences). The primary antibodies used were PE anti-human SSEA-4 (1:100; BioLegend), TRA-1-60 anti-human Vio 488 (1:100; Miltenyi Biotec), and Anti-Cardiac Troponin T (1:200; Richard-Allan Scientific). Secondary antibodies included Alexa Fluor 488 goat anti-mouse IgG (1:2000; Thermo Fisher Scientific).

### Differentiation in three germ layers

An in vitro differentiation assay was performed as described previously^42^. Briefly, D-HCM iPSCs suspension was seeded into 96U Bottom Plate (Thermo Fisher Scientific) and cultured with StemFit supplemented 10 µM Y-27632. To form an embryoid body (EB), the medium was changed to DMEM (Thermo Fisher Scientific) supplemented with 4 g/L D( +)-Glucose Solution (FUJIFILM Wako), 10% FBS, and 1% P/S (penicillin–streptomycin solution (FUJIFILM Wako)) every two days. After eight days, the EBs were transferred onto 0.1% gelatin-coated 12-well plates. After culturing for one week, immunostaining was performed and the differentiation potential into three germ layers was evaluated using a fluorescence microscope BZ-X710 (KEYENCE). The antibodies used were anti-mouse TUJ1 (1:100; R&D Systems), anti-mouse SMA (1:100; R&D Systems), anti-mouse AFP (1:100; R&D Systems), and Alexa Fluor 488 goat anti-mouse IgG (1:2000; Thermo Fisher Scientific).

### Transmission electron microscope: TEM

iPSC-CMs were seeded onto Matrigel-coated plates covered with a cover glass. Prefixation was performed with 2.5% glutaraldehyde/0.1 M phosphate buffer for 30 min at room temperature. Samples were post-fixed, embedded, and polymerized at the Medical Electron Microscopy Laboratory of the Department of Medicine, University of Tsukuba. The ultrathin sections were placed on copper grids, imaged using a transmission electron microscope JEM-1400 (JEOL), and analyzed using ImageJ^44,45^.

### Ca^2+^ imaging

Ca^2+^ imaging was performed as described in our previous paper^46^. Briefly, iPSC-CMs were seeded onto Matrigel-coated glass-bottom dishes. The day before measurement, the medium was replaced with Ca^2+^ imaging medium (Supplemental Table). Just before the measurements, cells were incubated with 5 µM Fluo 4 AM special packaging (Dojindo) for 10 min in a 37 °C incubator. The intracellular Ca^2+^ kinetics of iPSC-CMs were measured using line scans on a confocal laser microscope (ZEISS) and analyzed using ImageJ software. To measure the time to peak and 50% decay time, we chose records of Ca^2+^ transients in which no EAD/DAD appeared, and then measured the time from baseline to peak and the time from peak to 50% descent. After baseline recording, the samples were treated with 1 µM isoproterenol (iso) (β-receptor agonist, Kowa) and measured in the same way.

### Western blot

Western blotting was performed as described previously^42^. Briefly, proteins were extracted from the collected cells using the PRO-PREP Protein Extraction Solution (INB). Proteins were separated using sodium dodecyl sulfate–polyacrylamide gel electrophoresis (SDS-PAGE), transferred to PVDF membranes, and incubated in blocking buffer (0.1% Tris-buffered saline (Santa Cruz) supplemented with 3% skim milk (FUJIFILM Wako) and 0.1% polysorbate 20 (MP Biomedicals)) for one hour at room temperature. Next, the primary antibody diluted with blocking buffer was reacted overnight at 4 °C. The following day, the secondary antibody was diluted in the blocking buffer and reacted for one hour at room temperature. After washing, the target proteins were detected using the ELC Prime Western Blotting Detection Reagent (Cytiva) and photographed using the chemiluminescence imaging system FUSION FX7.EDGE (Vilber Bio Imaging). The ImageJ software was used for statistical analysis. The antibodies used were against MYBPC3 antibody (E-7) (1:1000; Santa Cruz Biotechnology), Troponin I antibody (4002S) (1:1000; Cell Signaling) and actin antibody (C4) (1:1000; Santa Cruz Biotechnology). The cMYBP-C protein levels were calculated by standardization with β-actin.

### Cell metabolic analyses

The Cell Mito Stress Test and Real-Time ATP Rate Assay were performed using a Seahorse XFp Extracellular Flux Analyzer (Agilent Technologies), according to the manufacturer’s instructions. Four to five days before the measurement, iPSC-CMs were seeded onto Matrigel-coated XFp plates. On the day of the measurement, the medium was replaced with Seahorse XF RPMI Medium. The measurement reagents were 1.5 µM Oligomycin (oxidative phosphorylation inhibitor), 1 µM FCCP (deconjugating agent), and 0.5 µM Rotenone/Antimycin A (electron transfer system inhibitors). Seahorse Waves software (Agilent Technologies) were used for all the analyses.

### Reverse transcription quantitative polymerase chain reaction: RT-qPCR

RT-qPCR was performed as previously described^46^. Briefly, total RNA was purified from cell pellets using the RNeasy Mini Kit (QIAGEN). A High-Capacity cDNA Reverse Transcription Kit (Thermo Fisher Scientific) was used to synthesize cDNA from each RNA sample. qPCR was performed using Quant Studio 5 (Thermo Fisher Scientific) and the standard curve method. *GAPDH* primer (Integrated DNA Technologies) was used as an internal control. *MYBPC3*, *GLUT4*, *NDUFB1*, *COX5B*, *UQCRB*, and *ATPIF1* (Integrated DNA Technologies) were used as target primers.

### RNA sequencing

RNA was purified from cell pellets using a RNeasy Mini Kit (QIAGEN). After the samples were prepared, RNA sequencing was performed at the Department of Sports Medicine of the Organization for Open Facility Initiatives of the University of Tsukuba^47^. NGS was performed using a NextSeq 500 System (Illumina), and NGS data were confirmed using CLC Genomics Workbench 22.0 software (QIAGEN). Gene Set Enrichment Analysis (GSEA) software was used for RNA sequencing analysis, using the Gene Ontology (GO) gene set^16^. A heatmap was drawn using Morpheus software (https://software.broadinstitute.org/morpheus/).

### Statistical analyses

Values for each group are presented as mean ± standard error. Two-tailed t-tests or Mann–Whitney U tests were used to test 2-group comparisons. *p* < 0.05 was considered statistically significant (ns > 0.05, * < 0.05, ** < 0.01, *** < 0.001, **** < 0.0001). All analyses were performed using GraphPad Prism software.

### Supplementary Information

Supplementary Information.

## Data Availability

All data generated and/or analyzed in the current study are available from the corresponding author upon reasonable request. The total RNA-Seq data as FASTQ files and an expression browser as table data have been deposited in the “Sequence Read Archive (SRA) (https://www.ncbi.nlm.nih.gov/sra; accessed on 3 Apr 2024)” under accession number: SRP499665.
