# Supplementary material for: Metabolic remodeling and calcium handling abnormality in induced pluripotent stem cell-derived cardiomyocytes in dilated phase of hypertrophic cardiomyopathy with MYBPC3 frameshift mutation
Source: Sci Rep. 2024 Jul 4;14:15422. doi: 10.1038/s41598-024-62530-0 (PMC11224225; doi:10.1038/s41598-024-62530-0)
Supplement: Supplementary file 1 — Supplementary Information. [file 41598_2024_62530_MOESM1_ESM.pdf]

Supplementary Table S1. Mediums and reagents

Medium Name	Component	Supplier	Product No.	Final Concentration
PBMC medium	HPC Expansion Medium DXF	PromoCell	C-28021	1x
	Cytokine Mix E	PromoCell	C-39890	100x
	IL-6	PeproTech	200-06	100 ug/mL
StemFit	StemFit AK02N	Ajinomoto	RCAK02N	1x
	Penicillin-streptomycin solution	FUJIFILM Wako	168-23191	100x
RPMI-	RPMI1640 (Roswell Park Memorial Institute 1640)	Thermo Fisher Scientific	11875-093	1x
	B-27 minus insulin	Thermo Fisher Scientific	A18956-01	50x
	L(+)-ascorbic acid	FUJIFILM Wako	012-04802	50 ug/mL
	Penicillin-streptomycin solution	FUJIFILM Wako	168-23191	100x
RPMI+	RPMI1640	Thermo Fisher Scientific	11875-093	1x
	B-27 supplement	Thermo Fisher Scientific	17504-001	50x
	L(+)-ascorbic acid	FUJIFILM Wako	012-04802	50 ug/mL
	Penicillin-streptomycin solution	FUJIFILM Wako	168-23191	100x
Lactate medium	DMEM (Dulbecco's Modified Eagle Medium), no glucose, no glutamine, no phenol red	Thermo Fisher Scientific	A14430-01	1x
	L-Lactic Acid	FUJIFILM Wako	129-02666	4 mM
	Penicillin-streptomycin solution	FUJIFILM Wako	168-23191	100x

Fatty acid medium	DMEM, no D-Glucose	Thermo Fisher Scientific	11966-025	1x
	HEPES	FUJIFILM Wako	342-01375	10 mM
	L-carnitine inner salt	Sigma-Aldrich	C0158	2 mM
	Taurine	FUJIFILM Wako	T007850	5 mM
	MEM (non-essential amino acids) Solution	Thermo Fisher Scientific	132-15641	100x
	Insuline, Transferine, Selenium Solution (ITS-G)	Thermo Fisher Scientific	090-06741	100x
	Linoleic Acid-Oleic Acid-albumin	Sigma-Aldrich	L9655	100x
	Penicillin-streptomycin solution	FUJIFILM Wako	168-23191	100x
10% FBS medium	DMEM, no D-Glucose	Thermo Fisher Scientific	11966-025	1x
	Fetal Bovine Serum	biowest	S1810-500	10x
	D(+)-Glucose Solution	FUJIFILM Wako	079-05511	1 g/L
	Penicillin-streptomycin solution	FUJIFILM Wako	168-23191	100x
Calcium imaging medium	FluoroBrite™ DMEM	Thermo Fisher Scientific	A1896701	1x
	Fetal Bovine Serum	biowest	S1810-500	10x
	L-Glutamine	Sigma-Aldrich	G2150	4mM
	Penicillin-streptomycin solution	FUJIFILM Wako	168-23191	100x

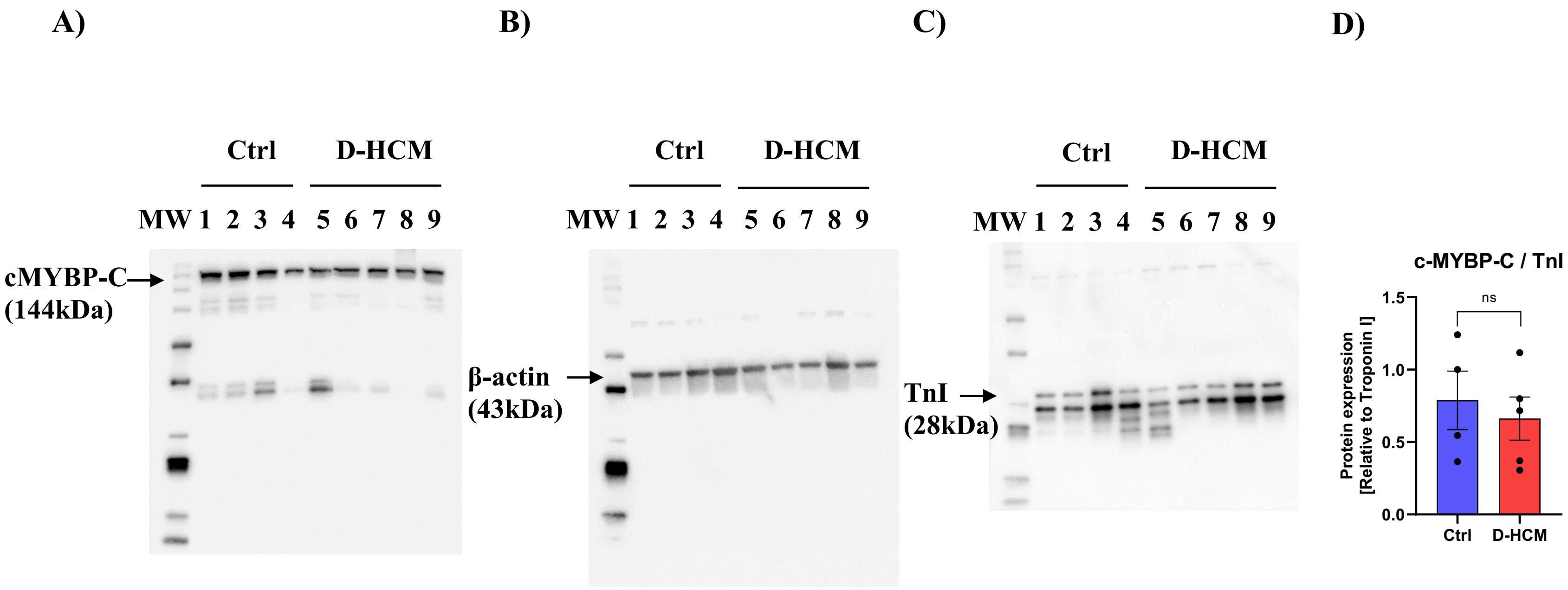

Supplementary Figure S1. Western blot analyses.
The whole images of western blots for A) cMYBP-C, B) β-actin, and C) Troponin I (TnI).
D) Protein expression level of c-MYBP-C / Troponin I.
